# Giving a Voice to Patients With Smell Disorders Associated With COVID-19: Cross-Sectional Longitudinal Analysis Using Natural Language Processing of Self-Reports

**DOI:** 10.2196/47064

**Published:** 2024-05-10

**Authors:** Nick S Menger, Arnaud Tognetti, Michael C Farruggia, Carla Mucignat, Surabhi Bhutani, Keiland W Cooper, Paloma Rohlfs Domínguez, Thomas Heinbockel, Vonnie D C Shields, Anna D'Errico, Veronica Pereda-Loth, Denis Pierron, Sachiko Koyama, Ilja Croijmans

**Affiliations:** 1 Institute of Medical Psychology and Behavioural Neurobiology University of Tübingen Tübingen Germany; 2 Department of Clinical Neuroscience Division of Psychology Karolinska Institutet Stockholm Sweden; 3 Centre d'Economie de l'Environnement Montpellier Centre National de la Recherche Scientifique, Institut National de Recherche pour l'Agriculture l'Alimentation et l'Environnement, Institut Agro Université de Montpellier Montpellier France; 4 Interdepartmental Neuroscience Program Yale University New Haven, CT United States; 5 Department of Molecular Medicine University of Padova Padua Italy; 6 School of Exercise and Nutritional Sciences San Diego State University San Diego, CA United States; 7 Department of Neurobiology and Behavior University of California Irvine, CA United States; 8 Department of Developmental and Educational Psychology University of Basque Country Leioa Spain; 9 Department of Anatomy Howard University College of Medicine Washington, DC United States; 10 Biological Sciences Department Fisher College of Science and Mathematics Towson University Towson, MD United States; 11 Goethe University of Frankfurt Frankfurt am Main Germany; 12 Laboratoire Évolution et Santé Orale Université Toulouse III Toulouse France; 13 Department of Medicine School of Medicine Indiana University Indianapolis, IN United States; 14 Language and Communication Department Faculty of Arts Radboud University Nijmegen Netherlands; 15 Centre for Language Studies Radboud University Nijmegen Netherlands

**Keywords:** parosmia, anosmia, phantosmia, hyposmia, hyperosmia, long-hauler, sentiment classification, web-based study, COVID-19, smell disorders

## Abstract

**Background:**

Smell disorders are commonly reported with COVID-19 infection. The smell-related issues associated with COVID-19 may be prolonged, even after the respiratory symptoms are resolved. These smell dysfunctions can range from anosmia (complete loss of smell) or hyposmia (reduced sense of smell) to parosmia (smells perceived differently) or phantosmia (smells perceived without an odor source being present). Similar to the difficulty that people experience when talking about their smell experiences, patients find it difficult to express or label the symptoms they experience, thereby complicating diagnosis. The complexity of these symptoms can be an additional burden for patients and health care providers and thus needs further investigation.

**Objective:**

This study aims to explore the smell disorder concerns of patients and to provide an overview for each specific smell disorder by using the longitudinal survey conducted in 2020 by the Global Consortium for Chemosensory Research, an international research group that has been created ad hoc for studying chemosensory dysfunctions. We aimed to extend the existing knowledge on smell disorders related to COVID-19 by analyzing a large data set of self-reported descriptive comments by using methods from natural language processing.

**Methods:**

We included self-reported data on the description of changes in smell provided by 1560 participants at 2 timepoints (second survey completed between 23 and 291 days). Text data from participants who still had smell disorders at the second timepoint (long-haulers) were compared with the text data of those who did not (non–long-haulers). Specifically, 3 aims were pursued in this study. The first aim was to classify smell disorders based on the participants’ self-reports. The second aim was to classify the sentiment of each self-report by using a machine learning approach, and the third aim was to find particular food and nonfood keywords that were more salient among long-haulers than those among non–long-haulers.

**Results:**

We found that parosmia (odds ratio [OR] 1.78, 95% CI 1.35-2.37; *P*<.001) as well as hyposmia (OR 1.74, 95% CI 1.34-2.26; *P*<.001) were more frequently reported in long-haulers than in non–long-haulers. Furthermore, a significant relationship was found between long-hauler status and sentiment of self-report (*P*<.001). Finally, we found specific keywords that were more typical for long-haulers than those for non–long-haulers, for example, fire, gas, wine, and vinegar.

**Conclusions:**

Our work shows consistent findings with those of previous studies, which indicate that self-reports, which can easily be extracted online, may offer valuable information to health care and understanding of smell disorders. At the same time, our study on self-reports provides new insights for future studies investigating smell disorders.

## Introduction

Chemosensory dysfunctions are among the distinguishing symptoms of COVID-19 [1-3]. Although many infected patients recover within weeks, a large percentage of patients experience long-term olfactory dysfunction even after recovery from the acute phase [4,5]. These individuals are known as smell long-haulers. Since smell impairment is often hard to notice and even harder to describe by patients (eg, patients commonly confuse smell disorders with taste disorders), information that can clarify the process of smell impairment or recovery can be useful to describe, understand, and track the phenomenon [6]. It is also unclear how distorted chemosensory perception relates to well-being or changes in behavior such as those related to food intake or avoidance.

Smell disturbances, categorized as quantitative (alterations in odor intensity) or qualitative (changes in perceived odor quality) [7,8], are common among COVID-19 long-haulers [1,9]. Symptoms can range from increased perceived intensity (hyperosmia) to faint perception of odors (hyposmia) or even complete loss of olfactory perception (anosmia). Qualitative changes may occur when the quality of the perceived odor is altered [7,8], which occurs, for instance, in case of parosmia (ie, distorted smell perception typically leading to perceived smells as bad odors, eg, the smell of food in good condition is perceived as garbage) or phantosmia (ie, perceiving smells without an olfactory stimulus, eg, smoke when there is no fire). Although these distortions are prevalent, individuals often lack technical terminology awareness, leading to challenges in accurate self-reporting and potential impacts on emotional well-being. Confusion may, for example, arise from terms such as “flavor” and “taste” [10], where individuals may report that they do not taste anything while only their smell is affected and not their taste. Similarly, different types of smell changes may cause confusion, as the quantity (complete or partial loss of smell) and quality of the changes indicate different impairments. Moreover, people are often inaccurate in reporting their olfactory performances, which may affect their emotional well-being and their awareness of olfactory dysfunction [11]. Properly classifying symptoms is crucial for clinical and research purposes, emphasizing the need for open-ended survey formats to capture nuanced experiences [12]. The difficulties in symptom description make it hard to capture a person’s particular olfactory experience in close-ended survey questions [9,13,14]. In our study, we employed open-ended surveys to collect comments [2], providing valuable insights like “smell is back to normal,” “my sense of smell fluctuates and is not as good as before,” or “the smell of coffee and onions has changed.”

The strong connection between smell and emotional-cognitive states involves neural pathways connecting the amygdala, thalamus, orbitofrontal cortex, and hippocampus [15-18]. Olfactory dysfunctions, often preceding neurodegenerative disorders, are evident in conditions such as Alzheimer disease, Parkinson disease, autism, and depression [16,19,20]. The detrimental effect of chemosensory dysfunction on emotional well-being is well-recognized [21-23] but is not fully understood with respect to olfaction in long-haulers. Patients have reported altered mental status as well as frustrations with COVID-19–related olfactory dysfunction [24]. Furthermore, olfactory dysfunction predicts the development of depression in adults [20]. Therefore, a negatively affected well-being or emotional tone when describing their symptoms would be expected in patients with COVID-19 and olfactory dysfunctions such as anosmia or parosmia, particularly in long-haulers.

In this study, we analyzed open-ended questions that were included in surveys conducted by the Global Consortium for Chemosensory Research (GCCR) [2,3]. As an initial validation of the informative value of the comments, a comparison was made between symptoms coded from open-ended comments and from the multiple-choice answers alone, administered during COVID-19 (survey 1) and after recovery (survey 2). This sets the stage to address the following 3 questions: (1) What are the frequencies of parosmia, phantosmia, and other olfactory dysfunctions (ie, hyposmia and anosmia) as reported in open-ended comments? (2) What is the well-being or emotional tone of people experiencing these symptoms as reported in open-ended comments? (3) What specific food-related experiences are related to these symptoms? Open-ended questions allow participants to voice their concerns that may not be covered by the other type of questions and are closer to how patients may report these symptoms to their general practitioner or health care worker [25]. The questions addressed smell loss while participants experienced COVID-19 (survey 1) and during a follow-up survey (survey 2). Analyzing these comments and their content contributes to better understanding, in a more ecologically valid way, of how long hauling might affect emotional well-being as it relates to olfactory experiences and the frequency and severity of symptoms compared with analyzing close-ended survey questions alone. On the basis of previously reported information, the following hypotheses have been formulated in this study.

1. Hypothesis aim 1: Recovery from smell loss is often accompanied by parosmia and phantosmia and is considered a sign of olfactory mucosa regeneration [1]. Considering that some smell-related symptoms may remain in COVID-19 long-haulers, we predict that long-haulers will have a greater occurrence of parosmia and phantosmia in addition to other potential chemosensory dysfunctions compared with non–long-haulers based on their own description of their olfactory symptom progression in survey 2.
2. Hypothesis aim 2: Using a machine learning aspect–based sentiment analysis, we predict that long-haulers will report significantly more emotional and psychological distress compared to non–long-haulers.
3. Hypothesis aim 3: We hypothesize that long-haulers reporting parosmia and phantosmia will exhibit avoidance behavior, resulting in omission of certain food and nonfood items. This will be apparent from a qualitative semantic analysis of the comments in survey 2.

## Methods

### Study Design

To investigate the study hypotheses, we used data previously available to the GCCR [2,3] that analyzed closed-ended responses. More specifically, we used data acquired by means of open-ended questions included in those surveys, not analyzed before in those studies. The data collection has been designed and planned following the structure of a prospective cohort study with cross-sectional characteristics, in which participants experiencing smell disorders completed a survey at timepoint 1 (survey 1) and were invited to complete a follow-up survey again at timepoint 2 (survey 2). Participants who had recovered at timepoint 2 were classified as non–long-haulers, whereas participants still experiencing smell disorders at timepoint 2 were classified as long-haulers. The differences between these 2 groups were analyzed and described.

### Study Participants

The initial survey (survey 1) was completed between April and September 2020 by 12,313 participants [2,3], of which 3386 participants also completed the follow-up survey (survey 2) at timepoint 2 (between September 2020 and February 2021). Participants self-selected to participate in survey 1. They were invited via email to participate in survey 2 if they previously agreed to be recontacted; provided an email address; completed survey 1 in English, Spanish, Italian, Dutch, or French; and reported a change in smell, taste, and flavor (via a symptom checkbox) in survey 1. Participants completed the second survey between 23 and 291 (median 200) days after the first survey. From this, data from 1560 participants were included in this study. The participants were classified as either non–long-haulers (n=673) or long-haulers (n=887) based on their self-reported smell ability at survey 2 relative to survey 1 (Table 1). We refer the reader to [1] for a detailed overview of the data collection. Dutch, French, Italian, and Spanish comments were translated to English for our analyses. Translations were conducted by native speakers.

**Table 1:** Sample characteristics of the second Global Consortium for Chemosensory Research web-based survey on COVID-19 that was administered globally between September 2020 and February 2021.

Demographics		Long-haulers (n=887)	Non–long-haulers (n=673)
Age (years), mean (SD)		43.8 (12.3)	43.9 (12.2)
Gender (female), n (%)		701 (79)	464 (68.9)
**Language, n (%)**			
	English	348 (39.2)	231 (34.3)
	Spanish	114 (12.9)	76 (11.3)
	Dutch	92 (10.4)	51 (7.6)
	Italian	70 (7.9)	34 (5.1)
	French	263 (29.7)	281 (41.8)

### Ethics Approval

This preregistered cross-sectional web-based study was approved by the Office of Research Protections of the Pennsylvania State University in the United States (STUDY00014904). This study was approved as an exempt study. The protocol complies with the revised Declaration of Helsinki and is compliant with privacy laws in the United States and European Union. Data reported here were collected from the GCCR core questionnaire. All participants provided an online consent to participate in the survey prior to proceeding with answering the survey questions. Only those participants who previously agreed to be recontacted and provided their emails were approached to complete the second survey. The study data were deidentified at the Pennsylvania State University before these were provided to other researchers for analysis. All participants volunteered to participate in this study, and no compensation was provided.

### Procedure

#### Aim 1: Occurrence of Smell Disorders in Long-Haulers

Our first aim was to determine the incidence of anosmia, hyposmia, phantosmia, and parosmia in long-haulers versus that in non–long-haulers by analyzing the free-text comments. At variance with [1], these complaints were categorized and counted based on descriptive comments and not on the participants’ self-reports by means of closed questions. The self-report question in [1] asking for changes in smell was meant to capture quantitative changes and was not always sensitive to capture individual experiences. The question that prompted the free-text comment was “Please describe any current changes in smell. Type ‘none’ if this is not applicable.”

The data were first processed by means of a concept-driven quantitative content analysis, as described by [26]. Comments recorded at both timepoints (n=2543) were coded manually by 8 different coders for the presence of anosmia, hyposmia, parosmia, phantosmia; whether the person indicated that they had recovered, not at all, partially or fully; and for mentioning food and nonfood odorous items according to a predetermined coding scheme (see coding scheme in Multimedia Appendix 1). Cases that did not meet any of the symptoms were excluded from further analyses. The responses that contained “none” entries (n=469) were also excluded from further analyses.

As an additional validation measure, the overlap between coded symptom prevalence of the free-text comments was compared to the self-reported prevalence on the multiple-choice question that was used in [1] (Multimedia Appendices 2-4). In these multiple-choice questions, participants were asked to rate whether they had noticed anosmia, hyposmia, parosmia, or phantosmia by asking the following questions, respectively: “I cannot smell at all/smells smell less strong than they did before my impairment” (anosmia/hyposmia), “smells smell different than they did before my impairment (the quality of smell has changed)” (parosmia), and “I can smell things that aren’t there (eg, I smell burning when nothing is on fire)” (phantosmia). Since multiple-choice questions for anosmia and hyposmia were not provided separately, we could only validate parosmia, phantosmia, as well as anosmia and hyposmia together.

#### Aim 2: Emotional Distress in Long-Haulers

For this aim, we trained a sentiment classification algorithm with LCF-ATEPC (local context focus–aspect term extraction and polarity classification) from PyABSA [27] (version 1.16.15; Heng Yang) on a data set of restaurant reviews available in PyABSA (Restaurant16) and then used the trained algorithm to first extract the so-called aspects, that is, keywords in each comment. Each aspect in each comment was classified as being negative, neutral, or positive (numerically coded as 1, 2, and 3, respectively). Comments such as “mustard smells really pungent, and I can’t be around it” or “I smell things a little less well” were classified as negative, while comments such as “I am back to normal in smelling” or “smell has come back completely after recovering” were classified as positive. To validate the results of the first model, we trained a second model with the same parameters on a data set of laptop reviews also available in PyABSA (Laptop14) and used that model to classify the comments and conduct the same analyses.

#### Aim 3: Salience of Food and Nonfood Items in Long-Haulers

Once the incidence and impact of smell disorders were established, we determined whether specific foods, drinks, or other objects were associated with long hauling, with the goal to examine whether specific items were more salient in long-haulers than in non–long-haulers. We first extracted all food and nonfood items from the comments that had been coded for aim 1. Qualitative relationships between items were visualized using word clouds. To quantify the use of specific words across groups, we then conducted a relative frequency analysis on the extracted food and nonfood words from comments.

### Statistical Analyses

#### Aim 1: Occurrence of Smell Disorders in Long-Haulers

We used logistic regressions to approach aim 1 (glm function with a binomial error structure of the stats package in R) and assessed whether the 2 categories of participants (long-haulers vs non–long-haulers) differed in terms of the reported disorders that they respectively experienced. The dependent variables were each of the 4 smell disorders studied, namely, parosmia, phantosmia, hyposmia, or anosmia (0 for absence, 1 otherwise). Our explanatory variable was “smell long-hauler status” (long-hauler vs non–long-haulers). We included age and gender of the participants and whether the comments were translated in English (0 for untranslated comments vs 1 for translated comments) as control variables as well as their interactions with the explanatory variable. We included all the main effects and interaction terms in the initial model, which were then simplified by removing the nonsignificant interaction terms to achieve the minimal adequate model. We centered “age” in all the models in order to make the effects more easily interpretable. Statistical analyses were performed using R software (version 4.1.3; R Foundation for Statistical Computing).

#### Aim 2: Emotional Distress in Long-Haulers

A chi-square test was conducted to examine the relationship between sentiment classifications and long-hauler status of the aspect-based sentiment classification algorithm. A mixed-effects logistic regression model was used to examine the relationship among sentiment, long-hauler status, and olfactory dysfunction type. All analyses were conducted with Python (version 3.8.8; Python Software Foundation) and the packages Scipy (version 1.9.3; Community library project) and Statsmodels (version 0.13.5; Jonathan Taylor).

#### Aim 3: Salience of Food and Nonfood Items in Long-Haulers

This aim was approached initially by creating word clouds and subsequently conducting a relative frequency analysis. For the word clouds, the extracted words describing food and nonfood objects were converted to lower case unigrams, bigrams, and trigrams by using the R package RWeka (version 0.4.44; Kurt Hornik) and TM (version 0.7.8; Ingo Feinerer). The R package wordcloud2 (version 0.2.1; Dawei Lang) and RColorBrewer (version 1.1.3; Erich Neuwirth) were used to create word clouds, where the frequency of an n-gram determines the size within the cloud. For the relative frequency analysis, we preprocessed and aggregated each group’s comments into a corpus based on the long-hauler status. Preprocessing steps included splitting the plain text comments into tokens. Tokens were lowercased, and numerals and punctuations were removed. Commonly used stop words were removed, and the text was lemmatized using Wordnet [28]. We then computed the frequency lists for each corpus in the comparison based on our preprocessed comments. The log-likelihood statistic was calculated for each word in the 2 frequency lists by constructing a contingency table based on word frequencies within and across corpora as per the method in [29]. Given that log-likelihood is a statistical significance measure, it does not compute the size of the difference between corpora; rather, it provides the words we have the most evidence for. Thus, to determine the influence of each word in each of the corpora, the relative frequency [30] method was used. By comparing the normalized frequencies for each word, this method returns a value (–1,1). In our case, 1 indicates that the word is overused in the long-hauler corpus, and –1 in the non–long-hauler corpus. Using these metrics, we determined the food and nonfood words in the corpora by manually coding words from each category with log-likelihood values greater than 3.84 (a significance threshold of *P*<.05 or lower) and selected for further analysis.

## Results

### Aim 1: Occurrence of Smell Disorders in Long-Haulers

The logistic regression examining the association between anosmia and long-hauler status could not be performed, as the number of participants reporting anosmia was too small in both long-haulers (17/750) and non–long-haulers (0/338; see Table 2; Multimedia Appendix 5). For the other smell disorders, the minimal adequate models that were run to obtain the results reported below were obtained by removing the nonsignificant interaction terms between smell long-hauler status and the 3 control variables (age, gender, and translation) in all models (.06<*P*<.98). The logistic regressions revealed a significant effect of the smell long-hauler status (long-hauler vs non–long-haulers) in terms of reported disorders (Multimedia Appendix 6). Long-hauler participants were significantly more likely to report symptoms interpreted as parosmia (β=.58, SE .14; *z*=4.06; odds ratio [OR] 1.78, 95% CI 1.35-2.37; *P*<.001; Multimedia Appendix 7) and hyposmia (β=.55, SE .13; *z*=4.16; OR 1.74, 95% CI 1.34-2.26; *P*<.001; Multimedia Appendix 8) compared to non–long-haulers. However, long-hauler status did not affect the likelihood to report symptoms associated with phantosmia (β=.09, SE .19; *z*=0.49; OR 1.10, 95% CI 0.76-1.62; *P*=.63; Multimedia Appendix 9). To obtain in-depth information on the relationship between smell disorders and the control variables (eg, sex, age), please consult Multimedia Appendices 6-9.

**Table 2:** Sample size of reported olfactory dysfunctions by smell long-hauling status and gender as coded from self-reports in the Global Consortium for Chemosensory Research survey on COVID-19 that was administered globally between September 2020 and February 2021.

Dysfunction		Long-hauler (female), n/N (%)	Non–long-hauler (female), n/N (%)
**Parosmia**			
	No dysfunction reported	331/430 (77)	165/238 (69.3)
	Dysfunction reported	68/122 (55.7)	266/298 (89.3)
**Phantosmia**			
	No dysfunction reported	497/637 (78)	196/294 (66.7)
	Dysfunction reported	100/113 (88.5)	37/44 (84.1)
**Hyposmia**			
	No dysfunction reported	233/286 (81.5)	114/176 (64.8)
	Dysfunction reported	364/464 (78.4)	119/162 (73.5)
**Anosmia**			
	No dysfunction reported	584/733 (79.7)	233/338 (68.9)
	Dysfunction reported	13/17 (76.5)	0

### Aim 2: Emotional Distress in Long-Haulers

The classifier that was trained on restaurant reviews was unable to classify the sentiment of 854 out of 1560 comments, and 838 out of 1560 could not be classified by the model trained on laptop reviews. These comments were removed from further analyses. There was a relationship between the classified sentiment and long-hauler status based on the model that was trained on restaurant reviews (*χ*^2^_2_=15.1; *P*<.001; Table 3) and laptop reviews (*χ*^2^_2_=30.3; *P*<.001).

In addition to comparing the sentiments of long-haulers and non–long-haulers, an additional analysis examined the effect of specific olfactory dysfunction on the comment’s sentiment. We therefore split the data set based on each smell disorder (Table 4; Multimedia Appendix 10) and compared how the sentiment changed when a smell disorder was reported versus when it was not reported. It is important to note that the split of the data was not exclusive, that is, participants who, for example, did not report parosmia, could still have reported hyposmia, phantosmia, or anosmia.

Within the comments of long-haulers, there was an effect of parosmia (β=–1.13, SE .35; OR 0.32, 95% CI 0.16-0.62; *P*=.001) and hyposmia (β=–.76, SE .33; OR 0.47, 95% CI 0.24-0.90; *P*=.02). No significant effects were found for phantosmia (β=–.53, SE .50; OR 0.59, 95% CI 0.19-1.45; *P*=.29) and anosmia (β=–.97, SE 1.10; OR 0.38, 95% CI 0.02-2.29; *P*=.38).

For the analysis of non–long-haulers’ comments, the variable anosmia was omitted because there were no comments on anosmia. In the non–long-hauler comments, there was no significant difference between the classified sentiments (Table 5; Multimedia Appendix 11). However, there was a significant effect for parosmia (β=–2.00, SE .47; OR 0.14, 95% CI 0.05-0.33; *P*<.001) and hyposmia (β=–1.59, SE .39; OR 0.20, 95% CI 0.09-0.43; *P*<.001) but not for phantosmia (β=–.01, SE .54; OR 0.99, 95% CI 0.32-2.78; *P*=.98).

As a validation of these results, the same analyses were conducted for the model that was trained on laptop reviews (Multimedia Appendix 12). The sentiment classification of long-hauler comments showed no significant effects for parosmia (β=–.22, SE .19; OR 0.80, 95% CI 0.56-1.15; *P*=.24), phantosmia (β=–.47, SE .28; OR 0.62, 95% CI 0.36-1.06; *P*=.08), hyposmia (β=.16, SE .20; OR 1.17, 95% CI 0.80-1.72; *P*=.43), or anosmia (β=.27, SE .62; OR 1.31, 95% CI 0.37-4.39; *P*=.66). For the non–long-hauler comments (Multimedia Appendix 13), a significant effect was found for parosmia (β=–.71, SE .30; OR 0.49, 95% CI 0.27-0.89; *P*=.02) and hyposmia (β=–.55, SE .28; OR 0.58, 95% CI 0.33-1.00; *P*=.05) but not phantosmia (β=.01, SE .51; OR 1.01, 95% CI 0.37-2.81; *P*=.99).

**Table 3:** Sample size (n=706) of classified sentiments by smell long-hauling status for the model trained on restaurant reviews. Responses are smell-related self-reports from a web-based survey on COVID-19 that was administered globally between September 2020 and February 2021.

Hauling status responses	Negative, n	Neutral, n	Positive, n
Responses from long-haulers	172	2	45
Responses from non–long-haulers	435	1	51

**Table 4:** Proportion of long-haulers’ comments and their sentiments, as classified by the model trained on restaurant reviews. The comments are smell-related self-reports from a web-based survey on COVID-19 that was administered globally between September 2020 and February 2021.

Dysfunction		Negative sentiment	Neutral sentiment	Positive sentiment
**Parosmia**				
	No dysfunction reported	0.44	0	0.07
	Dysfunction reported	0.46	0	0.03
**Phantosmia**				
	No dysfunction reported	0.76	0	0.09
	Dysfunction reported	0.13	0	0.01
**Hyposmia**				
	No dysfunction reported	0.33	0	0.05
	Dysfunction reported	0.56	0	0.06
**Anosmia**				
	No dysfunction reported	0.87	0	0.10
	Dysfunction reported	0.02	0	0

**Table 5:** Proportion of non–long-haulers’ comments and their sentiments, as classified by the model trained on restaurant reviews. The comments are smell-related self-reports from a web-based survey on COVID-19 that was administered globally between September 2020 and February 2021.

Dysfunction		Negative sentiment	Neutral sentiment	Positive sentiment
**Parosmia**				
	No dysfunction reported	0.45	0.01	0.17
	Dysfunction reported	0.34	0	0.03
**Phantosmia**				
	No dysfunction reported	0.72	0.01	0.17
	Dysfunction reported	0.07	0	0.03
**Hyposmia**				
	No dysfunction reported	0.36	0	0.15
	Dysfunction reported	0.42	0	0.06

### Aim 3: Salience of Food and Nonfood items in Long-Haulers

Word clouds were generated to visually explore differences in food and nonfood items mentioned differently in the comments of long-haulers and non–long-haulers (Multimedia Appendices 14 and 15). Superficially, the word clouds appeared similar for both groups. Non–long-haulers appeared to mention cheese, urine, and sweat somewhat more often than long-haulers. Both groups most often mentioned coffee, onion, and food, and for the nonfood items, they mentioned perfume and smoke.

The relative frequency analysis, reported in Table 6, revealed that lemon was mentioned more often by long-haulers, whereas wine, cheese, vinegar, and mustard were mentioned more often by non–long-haulers. For the nonfood items, long-haulers more often mentioned weird (presumably for weird smells), fire, gas, and eucalyptus among other smelling objects. This is in line with the finding that long-haulers more often report parosmia and thus report more of these foul-smelling objects in their comments, whereas non–long-haulers might report the objects that they can smell and taste again in their comments (eg, wine, cheese).

**Table 6:** Results of a relative frequency analysis showing a list of words that were reported significantly (*P*<.05) more often by long-haulers or non–long-haulers. These words were extracted from smell-related self-reports in a web-based survey on COVID-19 that was administered globally between September 2020 and February 2021.

		Log-likelihood	Occurrence^a^
**Food items**			
	Lemon	7.62	0.78
	Vinegar	9.90	–0.50
	Cheese	6.12	–0.52
	Wine	12.60	–0.63
	Mustard	4.22	–0.76
	Red wine	9.46	–0.86
**Nonfood items**			
	Weird	7.62	0.78
	Fire	7.62	0.78
	Gas	10.20	0.72
	Eucalyptus	4.75	0.71
	Detergent	6.14	0.64
	Air	9.14	0.60
	Scent	8.07	0.32
	Burning	4.90	0.27
	Smoke	4.05	0.24
	Thing	9.40	0.19
	Very	5.90	0.13
	Smell	4.08	0.03

^a^A larger occurrence value indicates that words were reported more frequently by long-haulers, and a lower (negative) value indicates a higher frequency for non–long-haulers.

## Discussion

### Principal Findings

First, our study shows that there is a strong relationship between long-hauler status and the incidence of both quantitative and qualitative smell changes, as apparent from our analysis of the open-ended comments. In addition, our analysis shows that there is a strong relationship between the smell disorder states as extracted from open-ended comments compared to those extracted from multiple-choice questions. Second, the sentiment analysis revealed that long-haulers are more negative in tone, underscoring the socioemotional impact of smell disorders on the individual. Third, we found that specific smell objects were mentioned in the free-text comments, differentiated by long-haulers and non–long-haulers, and specific smell dysfunction symptoms. We will elucidate these results more in the remainder of this discussion.

Our data show that, according to our first hypothesis, long-haulers reported a significantly higher occurrence of olfactory dysfunctions, in particular, parosmia (*P*<.001) and hyposmia (*P*<.001), than non–long-haulers did. This agrees with previous research that shows that olfactory dysfunctions, especially parosmia, appear to be part of long COVID [1]. This marks the need for accurately defining the sensory experience by introducing a clinical routine testing that may highlight the recovery pathway, even if partial, as well as to give feedback to patients on their clinical course. It also points toward a need for better sensory education among the population that often has no means of describing their perception accurately. Moreover, it is relevant that clinicians become aware of the relative inaccuracy of patients when reporting their chemosensory symptoms. Olfactory unawareness has been reported several times [31], in particular, in older and at-risk populations. However, even if reported to health care professionals, olfactory dysfunctions are seldom objectively tested [32]. Reduced or simplified tests may be of value [33,34]; yet, the clinical practice largely relies on self-reporting. In the case of patients with COVID-19, guided classification of odors revealed a perceptual landscape different from controls [35].

Our second aim was to link emotional distress to long-hauler status by using a machine learning model, thereby eliminating human bias. The sentiment classifier revealed a more negative tone in long-haulers’ comments, as has also been shown by other studies [36-38]. Clustering by symptoms indicated that parosmia or hyposmia was associated with more negative words for both long-haulers and non–long-haulers. This raises the question why this was not the case for phantosmia. The reason might be that parosmia and its stronger relationship with food intake results in, for example, loss of appetite [37,39] and therefore stronger emotional impact. However, ambiguity exists in the interpretation of these results as is also mentioned by [40], who argue that there is much room for improvement of machine learning models in the domain of health. The tendency of these results is interesting nonetheless, and follow-up analyses coupled to the analysis of food intake habits [41] that more specifically delves into the quality of life could shed a clearer light on the matter.

A semantic analysis of the objects mentioned within the comments suggested that the most frequently mentioned objects were similar for long-haulers and non–long-haulers concerning nonfood items (eg, perfume, smoke), while for food items, the 2 most frequent words were the same but switched between long-haulers and non–long-haulers. The word “onion” was used most frequently by non–long-haulers and “coffee” by long-haulers. Concerning differentially reported food words, “lemon” was mostly reported by long-haulers and “red wine” by non–long-haulers. When looking at the specific olfactory dysfunction, the largest problem seemed to arise in the nonfood words mentioned by participants with phantosmia. They seemed to often mention words such as “smoke” and “burning.” Objects reported by both groups may represent the most salient odor objects. Long-haulers may no longer smell these, while non–long-haulers may perceive them as the first smells they regain. However, aim 3 words were only analyzed on their occurrence and thus did not provide a means to examine whether their occurrence elicited positive or negative feelings. Exploring whether certain foods were avoided or approached during smell dysfunction and investigating compounds triggering parosmia could provide valuable indicators, such as pyrazines for coffee, disulfides for onion, thiols for garlic, and methoxypyrazines for wine [42].

The mentioning of words that describe a burning sensation in participants with phantosmia is consistent with those mentioned in previous findings [43]. It provides confirmation of these findings in a large sample and offers an overview of other words and sensations that may be associated with phantosmia, which may be helpful for clinicians. Although multiple hypotheses have been proposed on the causes of phantosmia, it may very well be that phantosmic sensations have varying causes [44]. Our data consist of a wide range of self-reports of these phantosmic sensations and are therefore suitable for a follow-up analysis on specific contexts that evoke a phantosmic sensation to further the understanding of phantosmic experiences and the mechanisms that underlie them. This also highlights the value of internet in medical research, which allows for a collection of large data sets on patients’ self-reports that may add to the understanding of olfactory dysfunctions.

The difficulty people face in discussing smells and olfactory experiences is a well-known phenomenon [11]. In this study, we analyzed open-ended comments to gain insight into the experiences of individuals experiencing olfactory dysfunctions that are not entirely understood or correctly named by patients experiencing these. First, we found a discrepancy between the information in the open-ended comments and the close-ended multiple-choice question that asks for olfactory dysfunction. This suggests that the comments contain valuable information that differs between groups and symptoms, highlighting the importance of this approach. This study emphasizes the importance of considering open-ended comments to gain a more holistic understanding of participants’ experiences and perceptions. The approach presented here, that is, manually coding short open-ended responses for different symptoms, may be used in combination with machine learning classification paradigms, to better understand patients’ concerns voiced in web-based settings such as in web-based survey research, online patient council groups, or by using brief digital notes from general practitioners as input. This additional source of information could lead to better identification of different diagnoses, and along the line, better understanding of the different types of smell disorders.

Finally, few people mentioned that months after developing COVID-19–induced smell dysfunction, their smell did improve, even to a level of functioning that was better than before the onset of COVID-19. We identified 13 cases of hyperosmia. It is currently unknown in what percentage this phenomenon occurs and what the mechanisms underlying this phenomenon are, and hyperosmia has been seldom reported after COVID-19 [45,46]. Speculatively, an increase in the awareness of smells after having lost one’s sense of smell for a brief period could potentially drive attentional experience with odors—in an “you don’t know what you’ve got until you lose it” way [47]. In the future, large-scale prevalence studies on smell disorders could investigate the percentage of people who report an improved sense of smell after recovering from a smell dysfunction.

We considered the validity of our analyses of open-ended responses, as opposed to the analyses of close-ended questions in [1]. Concurrent with their results, we found that long-haulers were more likely to experience parosmia and hyposmia, but in contrast to their findings for phantosmia, we did not find that long-haulers were more likely to experience phantosmia. This may result from the lack of overlap between our coding and participants’ self-reports. Generally, coders underestimated the prevalence of smell disorders. This effect was the highest for phantosmia, and may, therefore, have led to the discrepancy between [1] and our study. Possible explanations for the high underestimation of prevalence by coders are that participants did not report their whole experience in the comments or that participants overestimated their experiences in the multiple-choice questions. This is a downside to our approach, but overall, the overlap between the coded and self-reported smell disorders seemed to be in a good range and should therefore allow for a useful interpretation of the results that our natural language processing measures yielded.

### Limitations in Our Study

Our study has several limitations. There was a selection bias in the participant group. As in [1], we are aware that there might be a self-selection bias in completing these surveys, as participants experiencing severe symptoms may be more motivated to also complete the second survey. At the same time, participants who felt that they had severe smell disorders may select additional answer options on the multiple-choice questions and clarify their symptoms in the open-comment field. Thus, this selection bias, as compared with [1], could go either way. This provides an opportunity to compare both ways of asking about symptoms from participants, which we included in aim 1. Notwithstanding, we interpret the results with caution and phrase future suggestions and implications cautiously.

### Conclusion

The use of web-based surveys proved of value during COVID-19 since it allowed the rapid collection of data for monitoring infection-related chemosensory deficits. In addition to multiple-choice answers, a thorough description of symptoms could be extracted from open-ended answers. The surveys in this study made it possible to describe the qualitative changes in the chemosensory functions and explore their frequency. The distress of long-haulers could be investigated using a nonhuman unbiased algorithm for sentiment classification. Lastly, it was possible to highlight the different use of words related to food and nonfood items, possibly relating to the different perceptual experiences. In conclusion, we proved the validity of our approach, based on the analysis of open-ended questions to better understand the perceptual world of patients, described using their own words. Our analysis provides a new perspective on olfactory disturbances following COVID-19 that cannot be captured through closed-ended questions. On a more general level, this data science perspective can advance web-based survey-based patient research studies.

## Appendix

Multimedia Appendix 1 Guidelines that were used in aim 1 to code the comments regarding olfactory dysfunction and a description of the practical implementation.

Multimedia Appendix 2 Confusion matrix showing the agreement between the coders (open-ended comments) and the participants’ self-report (multiple-choice question) for parosmia (survey 1 and survey 2 combined).

Multimedia Appendix 3 Confusion matrix showing the agreement between the coders (open-ended comments) and the participants’ self-report (multiple-choice question) for phantosmia (survey 1 and survey 2 combined).

Multimedia Appendix 4 Confusion matrix showing the agreement between the coders (open-ended comments) and the participants’ self-report (multiple-choice question) for smell loss, that is, anosmia and hyposmia (survey 1 and survey 2 combined).

Multimedia Appendix 5 Frequency of self-reported olfactory dysfunctions by smell disorder long-haulers and non–long-haulers in the global web-based survey on COVID-19 conducted between September 2020 and February 2021.

Multimedia Appendix 6 Odds ratios from the logistic regressions examining the association between smell long-hauling and prevalence of olfactory disorders.

Multimedia Appendix 7 Logistic regression investigating whether smell long- vs. non-longhaulers differed in terms of reported parosmia they respectively experienced.

Multimedia Appendix 8 Logistic regression investigating whether smell long- vs. non-longhaulers differed in terms of reported hyposmia they respectively experienced.

Multimedia Appendix 9 Logistic regression investigating whether smell long- vs. non-longhaulers differed in terms of reported phantosmia they respectively experienced.

Multimedia Appendix 10 Proportion of long-haulers’ comments and their sentiments, as classified by the model trained on restaurant reviews. The comments are smell-related self-reports from a web-based survey on COVID-19 that was administered globally between September 2020 and February 2021.

Multimedia Appendix 11 Proportion of non–long-haulers’ comments and their sentiments, as classified by the model trained on restaurant reviews. The comments are smell-related self-reports from a web-based survey on COVID-19 that was administered globally between September 2020 and February 2021.

Multimedia Appendix 12 Sentiment classification from the model that was trained on laptop reviews. Showing the proportion of comments from long-haulers that were classified as negative, neutral, and positive across all smell disorders.

Multimedia Appendix 13 Sentiment classification from the model that was trained on laptop reviews. Showing the proportion of comments from non-long-haulers that were classified as negative, neutral, and positive across all smell disorders.

Multimedia Appendix 14 Word clouds of words that were extracted from long-hauler and non–long-hauler comments, where the size of each word is determined by its frequency.

Multimedia Appendix 15 Word clouds of words that were manually extracted from comments, grouped by olfactory dysfunction, where the size of the word represents its frequency.

## Abbreviations

GCCR: Global Consortium for Chemosensory Research
LCF-ATEPC: local context focus–aspect term extraction and polarity classification
OR: odds ratio

## Data Availability

The data sets and scripts generated and analyzed during this study are available in the Open Science Framework repository “Giving a voice to adults with COVID-19: An analysis of the open-ended comments from COVID-19 smell long-haulers and non-long-haulers on the Global Consortium for Chemosensory Research survey” [48].
