# Supplementary material for: Giving a Voice to Patients With Smell Disorders Associated With COVID-19: Cross-Sectional Longitudinal Analysis Using Natural Language Processing of Self-Reports
Source: JMIR Public Health Surveill. 2024 May 10;10:e47064. doi: 10.2196/47064 (PMC11127136; doi:10.2196/47064)
Supplement: Multimedia Appendix 1 [file publichealth_v10i1e47064_app1.pdf]

Coding scheme S1. Guidelines that were used in aim 1 to code the comments regarding their olfactory dysfunction.

- Every ‘smell change’ comment is coded by means of dummy coding in 5 smell change categories, each in one column:
    1. Parosmia (0: no; 1: yes)
    2. Phantosmia (0: no; 1: yes)
    3. Anosmia (0: no; 1: yes)
    4. Hyposmia (0: no; 1: yes)
    5. Hyperosmia (0: no; 1: yes)
    6. Recovered (0: no; 1: partial; 2: fully)
- Comments are also coded for mentioning of specific objects:
    7. Food and beverages (0: not mentioned; 1: present in comment)
    8. Non-food items (0: not mentioned; 1: present in comment)
- Coding in the present: at time of filling in the survey (i.e., ignore reports of previous changes in smell)

1. Parosmia
    - Mentioning that *something* (a source) smells different than before, generally worse than before, ‘it is like…’. Also: ‘everything smells like […]’
2. Phantosmia
    - Smelling something when a source is not present, smelling something that is not there, ‘it smells like […] (all the time)’.
3. Anosmia
    - ‘I can’t smell anything.’ ‘No smell at all’
4. Hyposmia
    - Smell is back to some extent (relative to before). ‘I can smell to some (lesser) extent’. ‘I can smell but smells/odors are faint’, or ‘smell comes and goes’ (morning/evening). ‘I can only smell [very specific real present object smell]’
5. Hyperosmia
    - Smell ability is better than before.
6. Recovered
    - Smell is back (2: yes). Smell is back to some degree/any recovery of smell (1: partial).
7. Some categories are mutually exclusive:

- Parosmia -> no anosmia
- Anosmia -> no hyposmia, recovery is always 0, Anosmia -> No parosmia
- Hyposmia -> no anosmia

8. No coding of item-specific anosmia (e.g. “I cannot perceive the smell of flowers and of sage”). These items should be commented as unsure, and can be discussed later.

Practical implementation of the coding scheme

Coders were first trained on the coding scheme by developing a set of rules, and a small subset of comments were coded followed by a discussion of comments that were difficult to code. Data was divided into eight roughly equal groups of around 350 comments. Groups were formed based on a semi-random number generator (draw 2542 times a number 1-8). A small set of comments (n=40), was coded by every coder to allow calculation of inter-coder agreement. Agreement between coders was calculated by means of Fleiss’s kappa. Agreement between the eight coders was deemed acceptable, for anosmia (κ=0.77), parosmia (κ=0.78), phantosmia (κ=0.58), hyposmia (κ=0.69), presence of food items (κ=0.80), and non-food items (κ=0.74). No agreement was reached on whether people recovered fully, partially or not at all (κ=0.13). Coders marked cases of which they were uncertain, and these were discussed during a group meeting. This discussion led to the inclusion of an additional variable, “hyperosmia”, or an *increased* sensitivity in the sense of smell. However, due to its low prevalence (n=13) none of the analyses included this variable. There were 169 cases that were unresolved, and these were again re-coded by two previous coders and two new coders. Unresolved comments from the two previous coders were exclusively analysed by the two new coders.
