# Supplementary material for: Giving a Voice to Patients With Smell Disorders Associated With COVID-19: Cross-Sectional Longitudinal Analysis Using Natural Language Processing of Self-Reports
Source: JMIR Public Health Surveill. 2024 May 10;10:e47064. doi: 10.2196/47064 (PMC11127136; doi:10.2196/47064)
Supplement: Multimedia Appendix 2 [file publichealth_v10i1e47064_app2.pdf]

**Table S2. Confusion matrix showing the agreement between the coders (open-ended comments), and the participants' self-report (multiple choice question) for parosmia (S1 and S2 combined)**. The cross-table shows how many comments were coded as indicating a certain smell disorder (Coder: 1) or not (Coder: 0), and the overlap with the participants' report of having a certain smell disorder (Self-report: 1) or not (Self-report: 0). All "None"-entries were excluded from the analysis.

| | Coder: 0 | Coder: 1 |
|---|---|---|
| Self-report: 0 | 1150 | 174 |
| Self-report: 1 | 448 | 314 |
