# Supplementary material for: Giving a Voice to Patients With Smell Disorders Associated With COVID-19: Cross-Sectional Longitudinal Analysis Using Natural Language Processing of Self-Reports
Source: JMIR Public Health Surveill. 2024 May 10;10:e47064. doi: 10.2196/47064 (PMC11127136; doi:10.2196/47064)

Figure S6. Odds ratios from the logistic regressions examining the association between smell long-hauling and prevalence of olfactory disorders. Long-hauler status: smell long- versus non-long-hauler (reference category), Gender: men versus women (reference category), Translation: yes versus no (reference category).

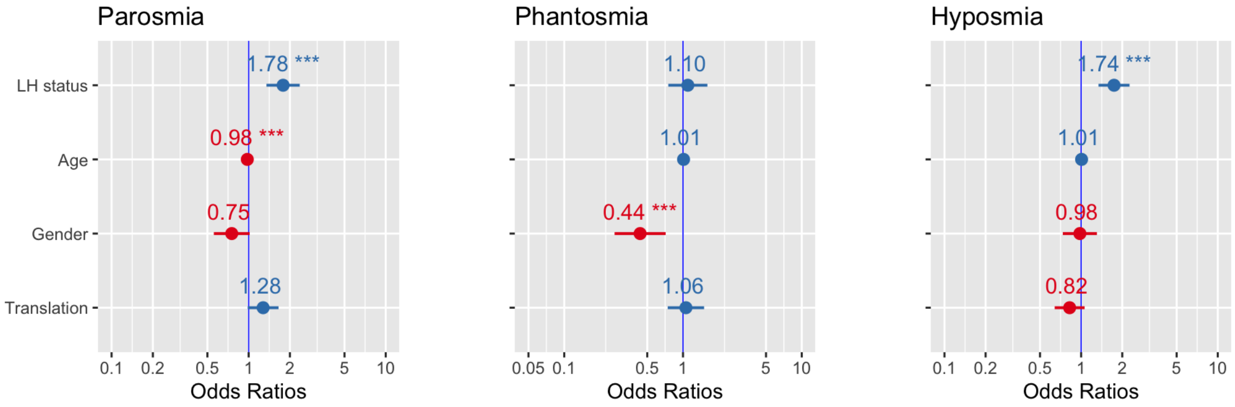

Supplement: Multimedia Appendix 6 [file publichealth_v10i1e47064_app6.pdf]
