# Supplementary material for: Giving a Voice to Patients With Smell Disorders Associated With COVID-19: Cross-Sectional Longitudinal Analysis Using Natural Language Processing of Self-Reports
Source: JMIR Public Health Surveill. 2024 May 10;10:e47064. doi: 10.2196/47064 (PMC11127136; doi:10.2196/47064)
Supplement: Multimedia Appendix 7 [file publichealth_v10i1e47064_app7.pdf]

Table S7. Logistic regression investigating whether smell long- vs. non-longhaulers differed in terms of reported parosmia they respectively experienced. For each variable, the estimate (β), the standard error of the mean (SE), the z statistic, and the p-value are given. The estimate of the variable *Longhauling status* is for the comparison between the longhaulers (reference category) and the non-longhaulers. The estimate of the variable *Gender* is for the comparison between men (reference category) and women. The estimate of the variable *Translation* is for the comparison between translated (reference category) or untranslated comments into English.

	ß	*SE*	z	*p*
Intercept	-0.97	0.15	-6.27	
Longhauling status	0.58	0.14	4.06	<0.0001
Age*	-0.02	0.005	-4.27	<0.0001
Gender	-0.28	0.15	-1.85	0.06
Translation	0.24	0.13	1.84	0.07

* The variable *Age* was centered.
