# Supplementary material for: Giving a Voice to Patients With Smell Disorders Associated With COVID-19: Cross-Sectional Longitudinal Analysis Using Natural Language Processing of Self-Reports
Source: JMIR Public Health Surveill. 2024 May 10;10:e47064. doi: 10.2196/47064 (PMC11127136; doi:10.2196/47064)
Supplement: Multimedia Appendix 8 [file publichealth_v10i1e47064_app8.pdf]

Table S8. Logistic regression investigating whether smell long- vs. non-longhaulers differed in terms of reported hyposmia they respectively experienced. For each variable, the estimate (β), the standard error of the mean (SE), the z statistic, and the p-value are given. The estimate of the variable *Longhauling status* is for the comparison between the longhaulers (reference category) and the non-longhaulers. The estimate of the variable *Gender* is for the comparison between men (reference category) and women. The estimate of the variable *Translation* is for the comparison between translated (reference category) or untranslated comments into English.

	ß	*SE*	z	*p*
Intercept	0.05	0.14	0.36	
Longhauling status	0.55	0.13	4.16	<0.0001
Age*	0.008	0.005	1.48	0.14
Gender	-0.02	0.15	-0.14	0.89
Translation	-0.19	0.13	-1.51	0.13

* The variable *Age* was centered.
