# Supplementary material for: Giving a Voice to Patients With Smell Disorders Associated With COVID-19: Cross-Sectional Longitudinal Analysis Using Natural Language Processing of Self-Reports
Source: JMIR Public Health Surveill. 2024 May 10;10:e47064. doi: 10.2196/47064 (PMC11127136; doi:10.2196/47064)
Supplement: Multimedia Appendix 9 [file publichealth_v10i1e47064_app9.pdf]

**Table S9. Logistic regression investigating whether smell long- vs. non-longhaulers differed in terms of reported phantosmia they respectively experienced.** For each variable, the estimate (β), the standard error of the mean (SE), the z statistic, and the p-value are given. The estimate of the variable *Longhauling status* is for the comparison between the longhaulers (reference category) and the non-longhaulers. The estimate of the variable *Gender* is for the comparison between men (reference category) and women. The estimate of the variable *Translation* is for the comparison between translated (reference category) or untranslated comments into English.

| | ß | *SE* | z | *p* |
|---|---|---|---|---|
| Intercept | -1.73 | 0.20 | -8.474 | |
| Longhauling status | 0.09 | 0.19 | 0.49 | 0.63 |
| Age* | 0.01 | 0.007 | 1.51 | 0.13 |
| Gender | -0.83 | 0.25 | -3.29 | 0.001 |
| Translation | 0.06 | 0.18 | 0.31 | 0.75 |

* The variable *Age* was centered.
