# Supplementary material for: Giving a Voice to Patients With Smell Disorders Associated With COVID-19: Cross-Sectional Longitudinal Analysis Using Natural Language Processing of Self-Reports
Source: JMIR Public Health Surveill. 2024 May 10;10:e47064. doi: 10.2196/47064 (PMC11127136; doi:10.2196/47064)

Figure S10. Proportion of long-haulers' comments and their sentiments, as classified by the model trained on restaurant reviews. The comments are smell-related self-reports from a web-based survey on COVID-19 that was administered globally between September 2020 and February 2021. 0: no dysfunction reported; 1: dysfunction reported.

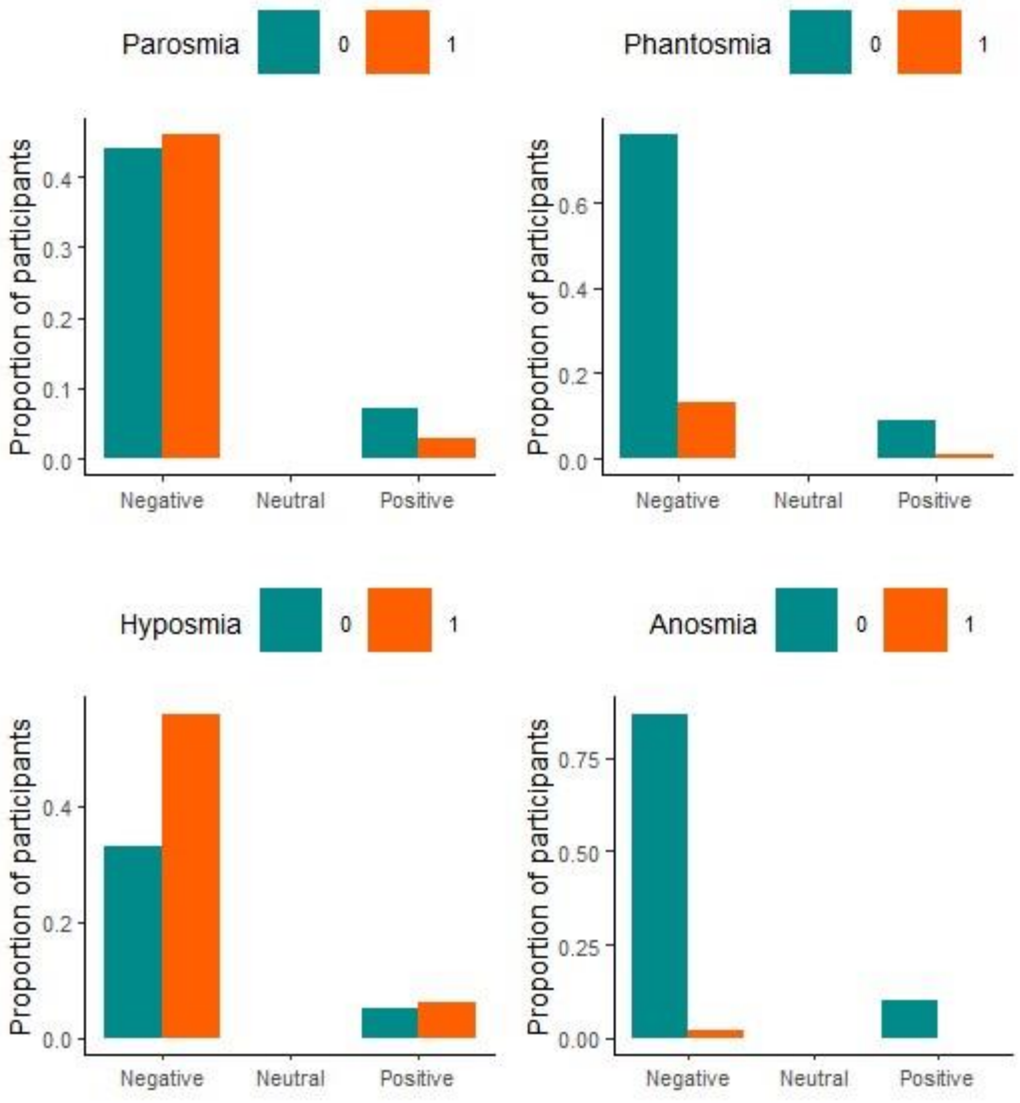

Supplement: Multimedia Appendix 10 [file publichealth_v10i1e47064_app10.pdf]
