# Supplementary material for: Giving a Voice to Patients With Smell Disorders Associated With COVID-19: Cross-Sectional Longitudinal Analysis Using Natural Language Processing of Self-Reports
Source: JMIR Public Health Surveill. 2024 May 10;10:e47064. doi: 10.2196/47064 (PMC11127136; doi:10.2196/47064)

Figure S13. Sentiment classification from the model that was trained on laptop reviews. Showing the proportion of comments from non-long-haulers that were classified as negative, neutral, and positive across all smell disorders. 0: No dysfunction reported, 1: Dysfunction reported.

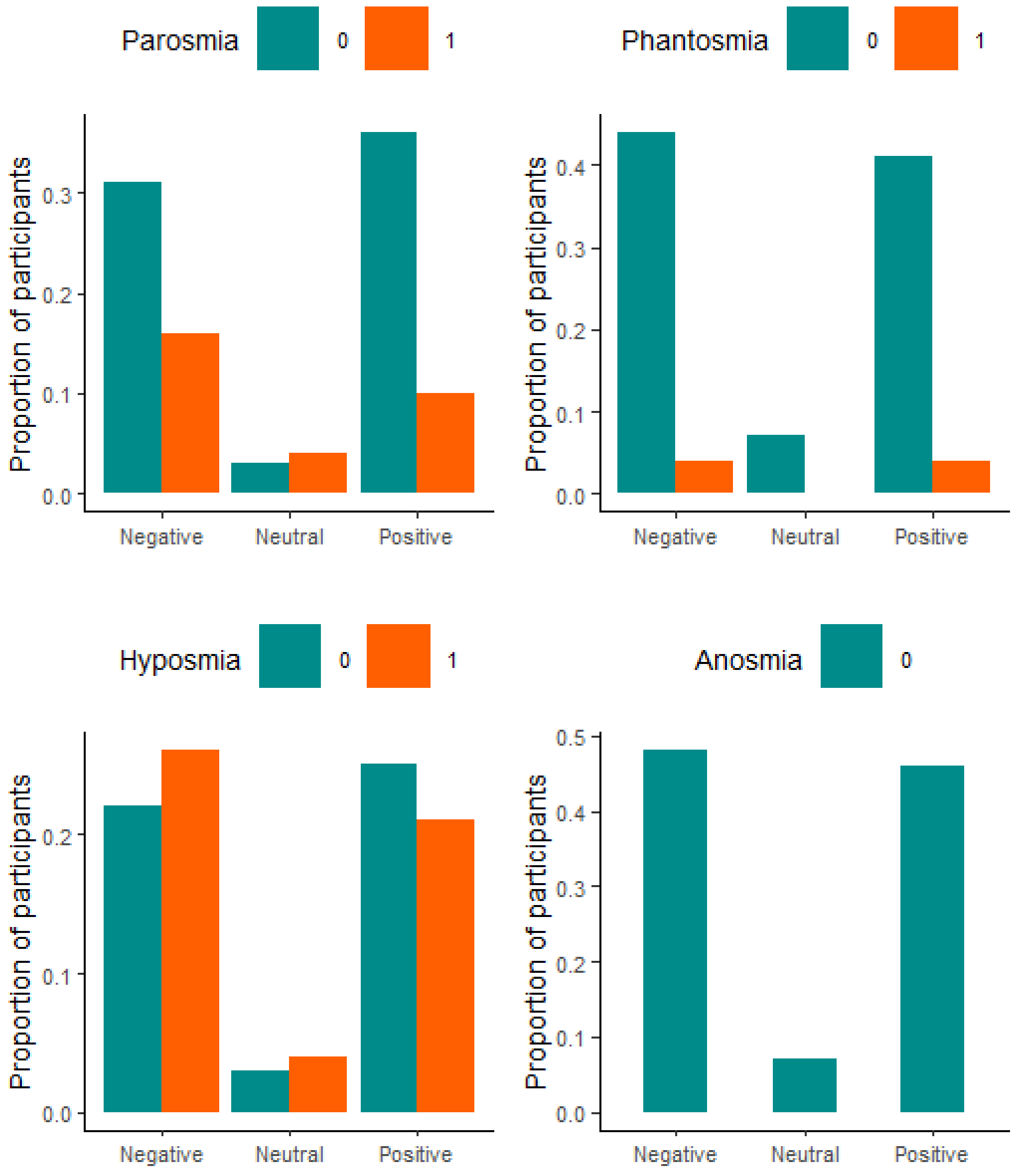

Supplement: Multimedia Appendix 13 [file publichealth_v10i1e47064_app13.pdf]
