# Supplementary material for: Giving a Voice to Patients With Smell Disorders Associated With COVID-19: Cross-Sectional Longitudinal Analysis Using Natural Language Processing of Self-Reports
Source: JMIR Public Health Surveill. 2024 May 10;10:e47064. doi: 10.2196/47064 (PMC11127136; doi:10.2196/47064)

Figure S14. Word clouds of words that were extracted from long-hauler and non–long-hauler comments, where the size of each word is determined by its frequency. The comments are smell-related self-reports from a web-based survey on COVID-19 that was administered globally from September to November in the fall of 2020.

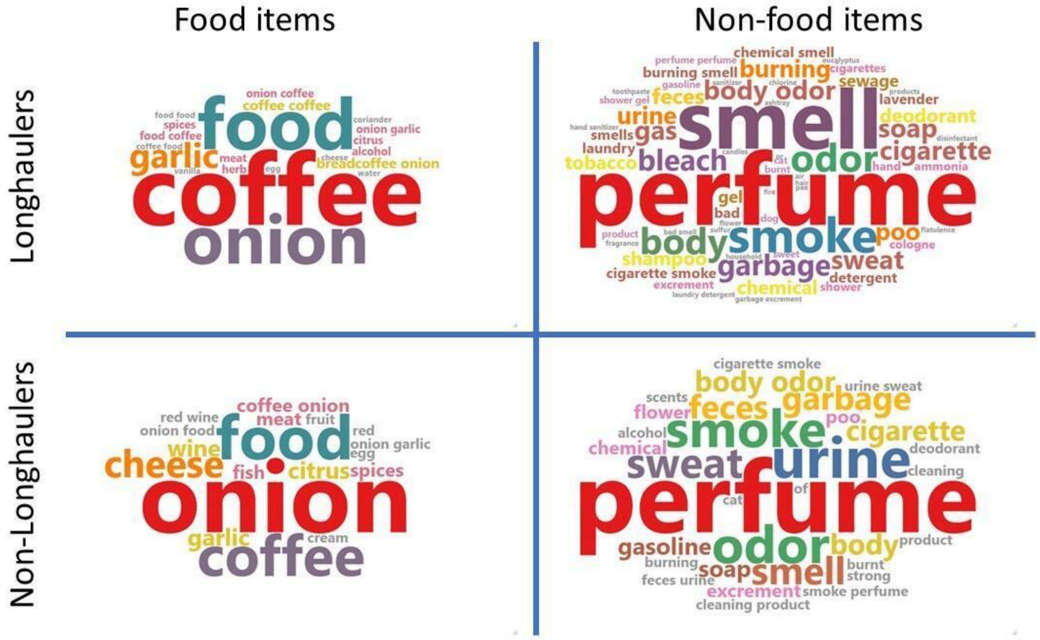

Supplement: Multimedia Appendix 14 [file publichealth_v10i1e47064_app14.pdf]
